# Editorial: Infections, reproductive health and non-communicable diseases

**DOI:** 10.4314/ahs.v25i4.1

**Published:** 2025-12

**Authors:** James K Tumwine

Welcome to this December 2025 issue of *African Health Sciences*. It is slimmer than the previous issue but it contains a very rich menu of original papers.

In the infectious disease section, we begin with an evaluation of procalcitonin for bacterial sepsis diagnosis^1^, followed by a treatise on the uptake and roll out of the WHO approved technologies for tuberculosis diagnosis in Africa^2^. It is followed by a study of biological quality of drinking water sources in Nigeria^3^.

While the HIV pandemic has remained an important infectious disease in our continent, there are a number of issues that keep it on the radar. One such issue is vitamin B12 levels^4^, as well as quality of life among persons receiving antiretroviral therapy^5^. Vaccines are in the news these days with numerous outbreaks of infectious childhood illnesses in high income countries. Barriers to vaccination of children^6^, and hepatitis B vaccination of medical workers concludes this section on infectious diseases^7^.

This leads us to papers on sexual reproductive health. First on the list is a paper by Odongo and colleagues^8^ on menopause, followed by one on use of modern contraceptives^9^. Then comes a paper on sexual reproductive health of refugees in Uganda^10^ spiced by one on experiences of teenage mothers^11^, and a report on adverse pregnancy outcomes in Ethiopia^12^. The section is capped with an interesting treatise on postpartum hemorrhages^13^, which is a very important cause of morbidity and mortality in Africa.

In the next section, non communicable diseases take center stage. Thus, we have papers on erythropoietic indices in asthma^14^, and pulmonary function among woodworkers exposed to hardwood dust^15^. Pericardiectomy for constrictive pericarditis is reported^16^. It is followed by papers on acute kidney injury among patients undergoing contrasted CT scan^17^, and hypertension in Eastern Uganda^18^. Mental health problems in the Kilimanjaro area of Northern Tanzania^19^, as well as post-traumatic stress disorder among displaced children in Nigeria^20^ cap this section on mental health issues.

We rarely cover health and human rights issues^21^. A treatise on patient satisfaction^22^ and determinants of nutrition status of children in a Nigerian community ends this Editor's choice^23^.

We thank our partners, authors, reviewers, readers, the editorial team and volunteers, without whose support, AHS would not have reached 25 years of publication. Have a fruitful reading and a happy festive season.
